# Prognostic value of three-dimensional ultrasound for fetal hydronephrosis

**DOI:** 10.3892/etm.2015.2168

**Published:** 2015-01-05

**Authors:** JUNMEI WANG, WEIWEN YING, DAXING TANG, LIMING YANG, DONGSHENG LIU, YUANHUI LIU, JIAOE PAN, XING XIE

**Affiliations:** 1 Department of Ultrasound, Women’s Hospital, School of Medicine, Zhejiang University, Hangzhou, Zhejiang 310006, P.R. China; 2 Department of Women’s Health, Women’s Hospital, School of Medicine, Zhejiang University, Hangzhou, Zhejiang 310006, P.R. China; 3 Department of Urology, Children’s Hospital, School of Medicine, Zhejiang University, Hangzhou, Zhejiang 310006, P.R. China; 4 Department of Ultrasound, Naval Convalescent Zone, Hangzhou Sanatorium, Nanjing Military Command, Nanjing, Jiangsu 310002, P.R. China; 5 Women’s Reproductive Health Laboratory of Zhejiang, School of Medicine, Zhejiang University, Hangzhou, Zhejiang 310006, P.R. China; 6 Department of Gynecological Oncology, Women’s Hospital, School of Medicine, Zhejiang University, Hangzhou, Zhejiang 310006, P.R. China

**Keywords:** three-dimensional ultrasound, fetal hydronephrosis, prognosis

## Abstract

The present study evaluated the prognostic value of three-dimensional ultrasound for fetal hydronephrosis. Pregnant females with fetal hydronephrosis were enrolled and a novel three-dimensional ultrasound indicator, renal parenchymal volume/kidney volume, was introduced to predict the postnatal prognosis of fetal hydronephrosis in comparison with commonly used ultrasound indicators. All ultrasound indicators of fetal hydronephrosis could predict whether postnatal surgery was required for fetal hydronephrosis; however, the predictive performance of renal parenchymal volume/kidney volume measurements as an individual indicator was the highest. In conclusion, ultrasound is important in predicting whether postnatal surgery is required for fetal hydronephrosis, and the three-dimensional ultrasound indicator renal parenchymal volume/kidney volume has a high predictive performance. Furthermore, the majority of cases of fetal hydronephrosis spontaneously regress subsequent to birth, and the regression time is closely associated with ultrasound indicators.

## Introduction

Fetal hydronephrosis, the incidence of which is between 0.17 and 2.3%, is one of the most common fetal abnormalities found in prenatal ultrasound examination (1). The majority of cases of fetal hydronephrosis spontaneously regress prior to or following birth, while only a few of them require further treatment postnatally (2); however, parents of the infants usually worry about the prognosis of the disease, particularly about whether postnatal surgery or the long-term use of antibiotics is required. It is, therefore, essential to investigate how to use ultrasound to assess objectively the degree of fetal hydronephrosis, and to predict the outcome, particularly the likelihood of surgery.

The possibility of postnatal treatment for fetal hydronephrosis is proportional to its severity (1–3). Shapiro *et al* (4) proposed a hydronephrosis index in 2008, which was used to quantify and objectively reflect changes due to hydronephrosis (5). The dataset used to evaluate the sample, however, was not large enough to explore the association between the index and the prognosis of fetal hydronephrosis. Three-dimensional ultrasound can accurately measure the volume of different structures, particularly objects with irregular shapes. Duin *et al* (6) reported in 2008 that six researchers measured the pelvis volumes of 15 fetuses with pyelectasis using three-dimensional ultrasound, which was suggested to be a reliable method due to its good reproducibility; however, this research was only a methodological discussion. To the best of our knowledge, there has been no reported clinical application of three-dimensional ultrasound for the measurement of pelvis volume or its application in assessing the degree and prognosis of fetal hydronephrosis. In the present study, the expanded renal pelvis volume and kidney volume were measured in 180 cases of fetal hydronephrosis using three-dimensional ultrasound, and the degree of hydronephrosis was quantitatively evaluated using renal parenchymal volume/kidney volume measurements.

## Materials and methods

### Patients

In the present study, 180 pregnant females with fetal hydronephrosis were examined in the Women’s Hospital, School of Medicine, Zhejiang University (Hangzhou, China) between January 2009 and October 2013. The enrolled patients were aged between 20 and 42 years with an average age of 28.56±4.47 years. The pre-pregnancy weights of the females were between 40 and 72 kg with an average weight of 56.33±7.37 kg, and the pre-pregnancy heights were between 142 and 173 cm with an average height of 159.18±4.61 cm.

The inclusion criteria were as follows: i) ≥28 weeks gestation; ii) pelvic anteroposterior diameter (APD) ≥10 mm; iii) singleton pregnancy; and iv) no other obvious abnormalities found by ultrasound examination during pregnancy with the exception of hydronephrosis. Maternal pre-pregnancy weight and height and the side of fetal hydronephrosis were recorded. Fetuses with hydronephrosis were excluded if they had other system malformations or chromosomal abnormalities. Written informed consent was obtained from all patients and the study was approved by the Ethics Committee of Zhejiang University.

### Three-dimensional ultrasound measurements

The three-dimensional power Doppler mode was started (Voluson^™^ 730 Expert and Voluson^™^ E8; GE Healthcare, Vienna, Austria) when two-dimensional image quality was adjusted to optimal and the kidney structure was clearly displayed. The three-dimensional volume was obtained in the absence of fetal movement, when the patients were required to hold their breath and avoid movement. The section showing the maximum fetal kidney was set as the starting section to acquire the volume. The depth and width were adjusted according to the size of the kidney so that it occupied approximately three quarters of the screen. The scanning angle varied according to gestational age. The volume included the entire kidney, and the B plane (coronal section) of the volume was checked subsequent to obtaining the three-dimensional volume. Two or three valid three-dimensional kidney volumes were saved for volume measurement selection for each fetus. The 4D View^®^ Vocal software (GE Kretztechnik, Zipf, Austria) was used to measure the volumes, and the average was calculated. The kidney volume and expanded renal pelvic volume of each kidney with hydronephrosis were measured.

### Main indicators for hydronephrosis

**Figure 1:**
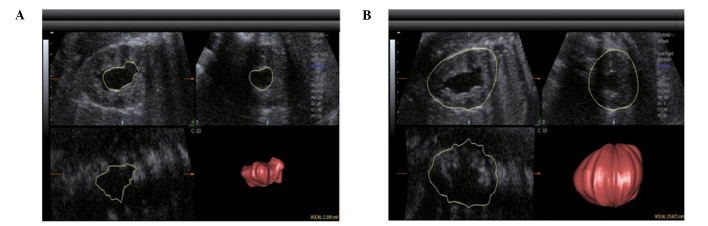
Ultrasound images showing (A) kidney volume V1 and (B) dilated renal pelvis volume V2. The renal parenchymal volume/kidney volume, (V1−V2)/V1 = (25.671–2.109)/25.671 = 0.92.

The following indicators for hydronephrosis were included: i) Pelvic APD (the maximum pelvic APD of the kidney cross-section); ii) the thickness of the renal parenchyma (the distance from the outermost edge of the renal collecting system on the long axis middle section to the outer edge of the kidney); iii) Society for Fetal Urology (SFU) grades (grade 0, no hydronephrosis; grade 1, mild renal pelvis dilatation; grade 2, mild calyx expansion with one or several calyces showing expansion; grade 3, expansion of all calyces; grade 4, calyx expansion with thinning of the renal parenchyma) (7); iv) hydronephrosis index (HI, %) [(total area of the kidney - area of the expanded renal pelvis)/total area of the kidney ×100 (5), which was measured on the largest hydronephrotic section on the long axis of the kidney]; and v) renal parenchymal volume/kidney volume [(total volume of the kidney - volume of the expanded renal pelvis)/total volume of the kidney] (Fig. 1).

### Postnatal follow-up of fetal hydronephrosis

Hydronephrosis was examined by ultrasound one week and one, three, six and 12 months after birth and reviewed every six months thereafter, until spontaneous regression of the hydronephrosis was observed. If hydronephrosis progressed rapidly, the time interval between the follow-ups was shortened. If the progression was rapid or the APD was ≥20 mm, radionuclide renography or excretory urography and magnetic resonance imaging examination were performed to determine the condition of the impaired renal function and the cause of the obstruction, prior to deciding whether surgery was required. Indications for surgery were as follows: i) APD >30 mm; ii) APD >20 mm and associated with calyx expansion; iii) split renal function <30%; iv) continued decline in renal function; v) continual increase in hydronephrosis; and vi) clear symptoms (3). The patients with spontaneous regression were followed up until hydronephrosis regression was observed. Patients who received surgery were followed up for pathological conditions and postoperative recovery.

### Statistical analysis

Data were analyzed using SPSS software (version 13; SPSS Inc., Chicago, IL, USA). A logistical regression method was used to analyze the association between fetal hydronephrosis outcome (whether to have surgery) and the side of hydronephrosis, the pelvic APD, renal parenchymal thickness, SFU grade, HI and the renal parenchymal volume/kidney volume value. Whether to have surgery for the fetal hydronephrosis was used as an outcome variable, the above-mentioned indicators were used as single diagnostic indicators, and a combination of selected different single indicators were used as comprehensive indicators to perform receiver operating characteristic (ROC) curve analyses. The ROC curves were plotted and the best prediction cutoff for each indicator was calculated. A two-sample t-test was used to compare the correlations between hydronephrosis side and regression time. Spearman rank correlation analysis was used to analyze the correlation between the APD, the HI, the SFU grade, renal parenchymal thickness, the renal parenchymal volume/kidney volume value and the regression time.

## Results

### Pathological conditions of fetal hydronephrosis

**Figure 2:**
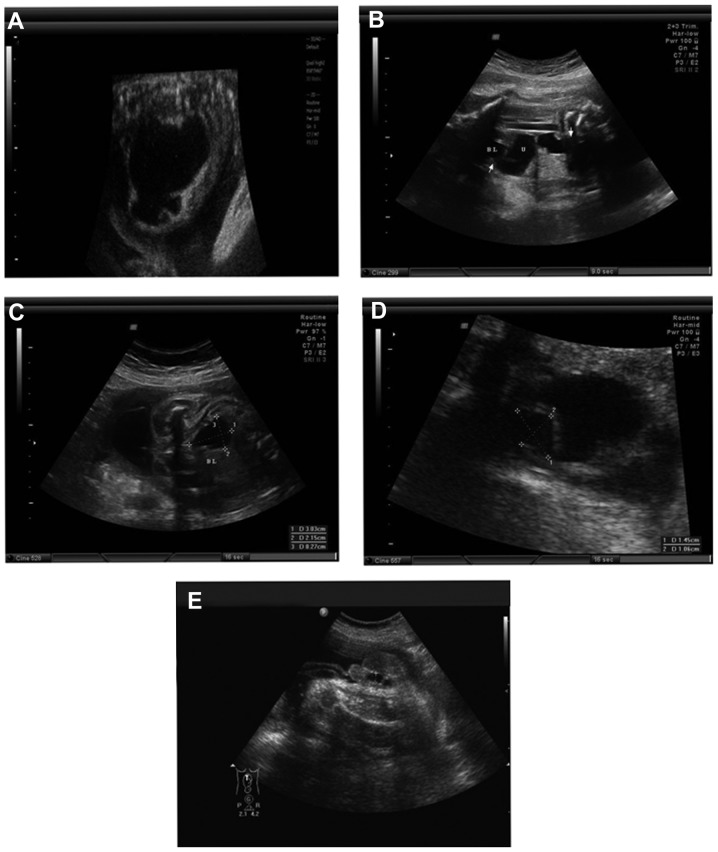
Ultrasound images of different pathological presentations of fetal hydronephrosis. (A) Pyelo-ureteral junction stricture showing pyelectasis. (B) Vesicoureteral reflux showing pelviureteric expansion. (C) Posterior urethral valves showing the typical keyhole sign. (D) Ureterocele showing bladder cyst. (E) Anterior urethral valves showing urethral expansion.

To examine the pathological condition of the fetal hydronephrosis of the patients, two-dimensional ultrasound was employed. In the 49 surgical cases, there were 35 cases of pyelo-ureteral junction stricture (Fig. 2A), seven cases of vesicoureteral reflux (Fig. 2B), three cases of posterior urethral valve (Fig. 2C), three cases of ureterocele (Fig. 2D) and one case of anterior urethral valve (Fig. 2E). With the exception of one case that had left kidney resection 10 months after birth due to severe left hydronephrosis, the post-operative follow-ups of the remaining surgical patients were good. In the 131 non-surgical patients, there were seven cases of vesicoureteral reflux, nine cases of extrarenal pelvis and 115 cases with no obvious pathological condition. A total of 110 cases exhibited spontaneous regression and 21 cases showed no significant changes in the degree of hydronephrosis during the follow-ups.

### Three-dimensional ultrasound can be used to predict whether fetal hydronephrosis requires surgery

To investigate the correlation between ultrasonic indicators and the outcome of fetal hydronephrosis, multivariate logistic regression analysis was performed using unilateral/bilateral hydronephrosis (unilateral, 0; bilateral, 1), the APD, the renal parenchymal thickness of the hydronephrotic side, the SFU grade, the HI and the renal parenchymal volume/kidney volume value as independent variables, and the hydronephrosis outcome (no surgery, 0; surgery, 1) as a dependent variable. Upon examination, unilateral/bilateral hydronephrosis, the APD and the renal parenchymal volume/kidney volume values were introduced into the model. Bilateral hydronephrosis, a larger APD or a lower renal parenchymal volume/kidney volume value suggested a greater risk of surgery (Tables I and II); therefore, a logistic regression model was established as follows: Logit (P) = 3.79 + 0.82 × unilateral/bilateral hydronephrosis + 1.05 × APD − 8.96 × renal parenchymal volume/kidney volume.

**Figure 3:**
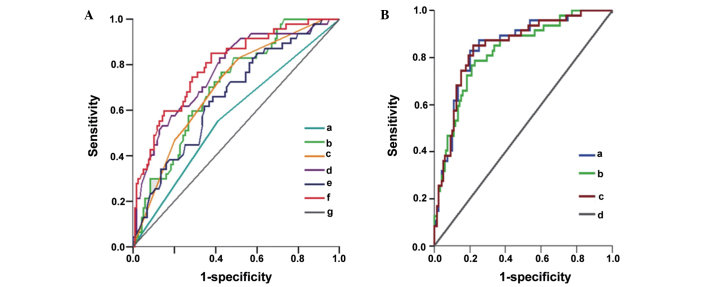
Receiver operating characteristic curves for the prediction of fetal hydronephrosis outcome. (A) Single indicator curve showing all the indices with the exception of lateral. a, Unilateral/bilateral hydronephrosis; b, hydronephrosis index; c, Society of Fetal Urology grade; d, pelvic APD; e, renal parenchymal thickness; f, renal parenchymal/kidney volume value; g, reference line. (B) Individual indicator combination curve showing that the efficiency of the combination of three indices is similar to that of the combination of six indices. a, Six indicators; b, pelvic APD + renal parenchymal volume/kidney volume value; c, unilateral/bilateral hydronephrosis + pelvic APD + renal parenchymal/kidney volume value; d, reference line. APD, anteroposterior diameter.

In addition, ROC curve analysis was performed using the requirement for surgery for the fetal hydronephrosis as the outcome variable (surgery, 1; no surgery, 0), and unilateral/bilateral hydronephrosis, the APD, the thickness of the renal parenchyma, the SFU grade, the HI and the renal parenchymal volume/kidney volume value as single diagnostic indicators (Table III). The results showed that the APD, the thickness of the renal parenchyma, the SFU grade, the HI and the renal parenchymal volume/kidney volume value, but not unilateral/bilateral hydronephrosis, had predictive significance for the requirement for postnatal surgery. The renal parenchymal volume/kidney volume value had the highest forecast accuracy, and the APD had the second highest (Table IV and Fig. 3A).

In addition, ROC curve analysis was performed using the combination of three indicators (unilateral/bilateral hydronephrosis, the APD and the renal parenchymal volume/kidney volume) or the combination of two indicators (the APD and renal parenchymal volume/kidney volume). The test results showed that the predictive accuracy of the combined three indicators was higher than that of the single indicators (Table V and Fig. 3B). When using 0.255 as the predictive possibility cutoff, the Youden index was the highest (0.64), with the sensitivity being 85.71% and the specificity being 78.46%.

Using unilateral/bilateral hydronephrosis, the APD, renal parenchymal volume/kidney volume and hydronephrosis outcome, the logistic regression model was established to give the following formula: P = e^3.786 + 0.822 × unilateral/bilateral hydronephrosis + 1.048 × APD − 8.955 × renal parenchymal volume/kidney volume^/1 + e^3.786 + 0.822 × unilateral/bilateral hydronephrosis + 1.048 × APD − 8.955 × renal parenchymal volume/kidney volume^.

According to this formula, the probability of fetal hydronephrosis outcome (surgery) could be calculated. A P-value ≥0.255 was judged as positive (surgery was required), while a P-value <0.255 was judged as negative (no surgery was required). Using this model to retrospectively evaluate the discrimination effect on the samples, the accuracy was 78.77%, the sensitivity was 81.63% and the specificity was 77.69%. These data demonstrated that ultrasound, particularly three-dimensional ultrasound, can be used to predict whether surgery is required for the treatment of fetal hydronephrosis.

### Three-dimensional ultrasound can be employed to predict the regression time of non-surgical cases subsequent to birth

To determine the correlation between fetal hydronephrosis indicators and the postnatal regression time of non-surgical cases, the association between any of the aforementioned six ultrasound indicators and the regression time of hydronephrosis was individually analyzed. The results showed that bilateral hydronephrosis had a longer regression time than unilateral hydronephrosis (t=−2.82, P=0.01). A larger APD, a higher SFU grade and a lower renal parenchymal volume/kidney volume value suggested a longer regression time (r=0.191, P=0.030; r=0.189, P=0.031; r=−0.293, P=0.001, respectively). The HI and the thickness of the renal parenchyma had no significant correlation with the regression time of hydronephrosis (r=−0.082, P=0.358; r=−0.144, P=0.155). These data indicated that three-dimensional ultrasound could predict the regression time of non-surgical cases following birth.

## Discussion

Studies by Ismaili *et al* (8) and Thornburg *et al* (9) indicated that ultrasound in late pregnancy could better predict whether to perform surgery for fetal hydronephrosis compared with that in mid-term pregnancy. Ismaili *et al* (8) reported that the positive predictive value of a late pregnancy APD of >0.7 cm was 69%, while that of a mid-term pregnancy APD of >0.4 cm was only 49%, since the majority (80%) of mid-term pregnancy cases of fetal hydronephrosis, particularly mild hydronephrosis, would regress in late pregnancy or following birth (10,11). A pelvic APD <1 cm usually means there is no disease (2,12–17). Woodward *et al* (2) reported that, in cases with an APD of <1 cm, only 3% had a deformity. In the study by Woodward *et al*, fetuses aged ≥28 weeks with hydronephrosis and an APD of ≥1 cm were studied. Using six indicators, namely unilateral/bilateral hydronephrosis, the APD, the thickness of the renal parenchyma, the SFU grade, the HI and the renal parenchymal volume/kidney volume value, the association between each indicator and the requirement for postnatal surgery was analyzed. The results showed that all six indicators could predict whether to perform postnatal surgery for the fetal hydronephrosis. Bilateral hydronephrosis, a larger APD, a thinner renal parenchyma, a higher SFU grade, a lower HI and a lower renal parenchymal volume/kidney volume value suggested a greater possibility of surgery. The cutoff for each indicator used to predict the possibility of surgery was obtained using the ROC curve. If the APD was >1.35 cm, the thickness of the renal parenchyma was <0.5 cm, the SFU grade was >2, the HI was <0.69 and the renal parenchymal volume/kidney volume value was <0.81, then a significant possibility of surgery was suggested. In the present study, a novel three-dimensional indicator, the renal parenchymal volume/kidney volume value, was introduced for the first time; this indicator had the best predictive performance, with the largest area under the ROC curve (0.8) and the highest Youden index (0.47). This could be associated with the high accuracy of three-dimensional ultrasound in measuring the expanded renal pelvis volume and kidney volume, which led to objective evaluation of the degree of hydronephrosis.

Using combined indicators to predict fetal hydronephrosis outcome usually has a higher accuracy than using single indicators. Zhan *et al* (18) used 0–3 points to score the APD, the thickness of the renal parenchyma and the renal pelvis shape according to the degree of hydronephrosis, and found that, when the combined score of the three indicators equaled ≤3, ≤4, ≤5, ≤6, ≤7 and ≥8, the proportions of pathological hydronephrosis were 0, 11.11, 28.57, 50.00, 80.00 and 100%, respectively. The best cutoff value to diagnose pathological fetal hydronephrosis was six, with the sensitivity and specificity being 88.46 and 94.49%, respectively. The present study indicated that combined indicators were more accurate than single indicators in predicting whether to perform surgery, and the area under the ROC curve for the combination of the six indicators was 0.85. Multivariate regression analysis showed that the accuracy of the combination of three indicators, unilateral/bilateral hydronephrosis, the APD and the renal parenchymal volume/kidney volume value, was close to that of the combination of six indicators, sharing similar 95% confidence intervals (0.78–0.91). The surgery possibility calculated using the logistic regression model constructed by the three indicators had an accuracy of 78.77%, a sensitivity of 81.63% and a specificity of 77.69%, using a P-value of 0.255 as the cutoff. The results suggested that the combination of the three indicators had a higher accuracy in predicting the possibility of postnatal surgery for fetal hydronephrosis.

To the best of our knowledge, only a few studies have reported the correlation between the degree of fetal hydronephrosis and postnatal regression time; these studies found that the lower the APD, the shorter the spontaneous regression time (19–21). The present study comprehensively analyzed the correlation between whether hydronephrosis could regress and each indicator, and the correlation between regression time and each indicator. The results showed that bilateral hydronephrosis, a larger APD, a higher SFU grade or a smaller renal parenchymal volume/kidney volume value indicated a longer spontaneous regression time, suggesting that more severe hydronephrosis was associated with a longer regression time. Among the indicators, the renal parenchymal volume/kidney volume value was the most relevant, suggesting a high application value for predicting whether spontaneous regression of fetal hydronephrosis would occur.

## Figures and Tables

**Table I:** Multivariate logistic regression analysis of each indicator and hydronephrosis outcome.

Variables	β	SE	Wald	*v*	P-value	OR (95% CI)
Unilateral/bilateral hydronephrosis	0.91	0.43	4.60	1.00	0.03	2.50 (1.08–5.76)
HI	0.51	2.68	0.04	1.00	0.85	1.67 (0.01–318.09)
SFU grade	0.12	0.43	0.08	1.00	0.78	1.13 (0.49–2.59)
APD	1.06	0.54	3.91	1.00	0.05	2.89 (1.01–8.28)
Thickness of the renal parenchyma	0.37	1.63	0.05	1.00	0.82	1.44 (0.06–34.98)
Renal parenchymal volume/kidney volume	−9.25	2.88	10.29	1.00	<0.01	0.00 (0.00–0.03)
Constant	2.20	3.44	0.41	1.00	0.52	

OR, odds ratio; CI, confidence interval; HI, hydronephrosis index; SFU, Society of Fetal Urology; APD, anteroposterior diameter; SE, standard error; *v*, degrees of freedom. The constant term represents when exposure dose of risk factors is 0.

**Table II:** Logistic regression analysis of three indicators and hydronephrosis outcome.

Variables	β	SE	Wald	*v*	P-value	OR (95% CI)
Unilateral/bilateral hydronephrosis	0.82	0.41	4.09	1.00	0.04	2.27 (1.03–5.04)
APD	1.05	0.49	4.53	1.00	0.03	2.85 (1.09–7.48)
Renal parenchymal volume/kidney volume	−8.96	2.37	14.32	1.00	<0.01	0.00 (0.00–0.01)
Constant	3.79	2.31	1.64	1.00	0.20	

OR, odds ratio; CI, confidence interval; APD, anteroposterior diameter; SE, standard error; *v*, degrees of freedom.

**Table III:** Receiver operating characteristic curve analysis of each indicator and fetal hydronephrosis outcome.

Variables	Area under the curve (95% CI)	P-value
Unilateral/bilateral hydronephrosis	0.57 (0.47–0.67)	0.156
HI	0.71 (0.62–0.79)	<0.001
SFU grade	0.70 (0.62–0.79)	<0.001
APD	0.77 (0.69–0.85)	<0.001
Thickness of renal parenchyma	0.66 (0.57–0.75)	0.001
Renal parenchymal volume/kidney volume	0.80 (0.72–0.87)	<0.001

CI, confidence intervals; HI, hydronephrosis index; SFU, Society of Fetal Urology; APD, anteroposterior diameter.

**Table IV:** Best predictive cutoff values of each single indicator.

Indicators	Best predictive cutoff value	Sensitivity (%)	Specificity (%)	Youden index
HI	0.69	82.98	51.26	0.34
SFU grade	2.00	69.52	57.05	0.27
APD	1.35	82.98	57.98	0.41
Thickness of renal parenchyma	0.50	72.34	52.94	0.25
Renal parenchymal volume/kidney volume	0.81	85.11	62.18	0.47

HI, hydronephrosis index; SFU, Society of Fetal Urology; APD, anteroposterior diameter.

**Table V:** Receiver operating characteristic curve analysis of the combinations of indicators and hydronephrosis outcome.

Variables	Area under the curve (95% CI)	P-value
Combination of six indicators	0.85 (0.78–0.91)	<0.001
Unilateral/bilateral hydronephrosis + APD + renal parenchymal/kidney volume	0.84 (0.78–0.91)	<0.001
APD + renal parenchymal/kidney volume	0.82 (0.76–0.89)	<0.001

CI, confidence interval; APD, anteroposterior diameter.
